# Calculation of Theoretical and Empirical Nutrient N Critical Loads in the Mixed Conifer Ecosystems of Southern California

**DOI:** 10.1100/tsw.2007.65

**Published:** 2007-03-21

**Authors:** Joan Breiner, Benjamin S. Gimeno, Mark Fenn

**Affiliations:** ^1^ USDA Forest Service, Pacific Southwest Research Station, 4955 Canyon Crest Dr., Riverside, CA 92507, USA; ^2^ Center for Conservation Biology, University of California, Riverside, CA 92521, USA; ^3^ Ecotoxicology of Air Pollution, CIEMAT (ed. 70), Avda. Complutense 22, 28040 Madrid, Spain

**Keywords:** critical loads, nitrogen leaching, Mediterranean ecosystems, simple mass balance, soil acidification

## Abstract

Edaphic, foliar, and hydrologic forest nutrient status indicators from 15 mixed conifer forest stands in the Sierra Nevada, San Gabriel Mountains, and San Bernardino National Forest were used to estimate empirical or theoretical critical loads (CL) for nitrogen (N) as a nutrient. Soil acidification response to N deposition was also evaluated. Robust empirical relationships were found relating N deposition to plant N uptake (N in foliage), N fertility (litter C/N ratio), and soil acidification. However, no consistent empirical CL were obtained when the thresholds for parameters indicative of N excess from other types of ecosystems were used. Similarly, the highest theoretical CL for nutrient N calculated using the simple mass balance steady state model (estimates ranging from 1.4–8.8 kg N/ha/year) was approximately two times lower than the empirical observations. Further research is needed to derive the thresholds for indicators associated with the impairment of these mixed conifer forests exposed to chronic N deposition within a Mediterranean climate. Further development or parameterization of models for the calculation of theoretical critical loads suitable for these ecosystems will also be an important aspect of future critical loads research.

## INTRODUCTION

The anthropogenic use of fossil fuels and agricultural fertilizers has increased the formation of reactive N species (Nr) by 90% during the last 150 years with continued growth in the emission rate expected over the next 50 years[1]. These Nr species induce environmental changes as they transfer through or are stored within each ecosystem, a process referred to as the N cascade[2], resulting in impacts on ecosystems that may ultimately impact human health adversely.

As a result, large-scale control policies aimed at reducing anthropogenic N emissions have been proposed in different regions of the world. One of the most successful internationally coordinated initiatives to control emissions has been undertaken by the United Nations – Economic Commission for

Europe (UNECE) Convention on Long-Range Transboundary Air Pollution (CLRTAP). Effect-oriented protocols for controlling specific pollutants have been proposed by using the critical loads/critical levels (CL) concepts or thresholds above which potential damage to different receptors may occur[3]. These concepts and the practicalities of their usage have been challenged[4], but they have proved to be useful tools for pollution abatement policy. In fact, the last European Union (EU) Environmental Action Programmes have applied these concepts and it is expected that, in the long-term, CL will not be exceeded anywhere in the EU. In contrast, in the U.S., CL have been little used for policy development, but interest in their use has increased in recent years, particularly within the context of protecting national parks and Class I wilderness areas and other federal lands[5].

The most straightforward and conceptually simple estimation of nutrient N CL for terrestrial ecosystems is the Simple Mass Balance (SMB) model[3], which assumes steady-state ecosystem conditions. The rationale behind this method is that $NO_3^-$ leaching occurs when N inputs exceed the N retention capacity of soil-plant-microbial systems, indicating N saturation of the ecosystem[6]. The SMB model assumes that detrimental effects on ecosystems arise when $NO_3^-$ leaching exceeds a given threshold. However, considerable disagreement has been found between the theoretical CL resulting from SMB calculations and those derived either empirically[7] or through ecosystem-process modeling[8]. It has been acknowledged that the exceedance of CL does not necessarily provide information about the actual eutrophication or acidification processes that could be occurring in the ecosystems being evaluated[9].

These discrepancies may be greater for Mediterranean ecosystems due to the particular features occurring in their distribution area such as: (a) the predominance of photochemical processes that transform airborne N molecules into phytotoxic substances such as ozone ($O_3$) or nitric acid ($HNO_3$) vapor[10]; (b) the prevalence of dry deposition over wet deposition[11,12] that, during subsequent precipitation events, may result in large pulses of N inputs to the soil with the solubilization of accumulated N deposited to plant and soil surfaces during long dry periods[13]; (c) the frequent temporal asynchrony between these large N input pulses and plant and microbial demand[14]; (d) high base cation saturation that favors increased nitrification with chronic N deposition[15]; and (e) the high incidence of forest fires that can result in volatilization of large amounts of N from the ecosystem, preventing it from reaching a steady state[16]. As a result of these peculiarities and in spite of being N limited, these ecosystems may be prone to $NO_3^-$ leaching in the early stages of their N-saturation[15], is in contrast with the mesic forests represented in the Aber et al. conceptual model[6] that predicts $NO_3^-$ leaching in the later stages of N saturation.

Therefore, an evaluation of the performance of the SMB model in relation to N cycling in Mediterranean ecosystems is needed as it is likely that its present formulation and the concepts behind it are not entirely suitable for these ecosystems. This has significant policy implications because incorrect CL for N could be established, leading to an erroneous risk assessment and, therefore, to potentially ineffective emission control policies or to misguided forest management practices. The well-documented N deposition gradient occurring in the mixed conifer forests of the Sierra Nevada, San Gabriel Mountains, and San Bernardino National Forest[12,17] provides a useful dataset to compare the theoretical N CL calculated with the SMB model and the empirical CL derived from the analysis of actual measurements of environmental parameters that have been proposed as indicators of ecosystem N status[18].

## MATERIALS AND METHODS

### Site Locations

Fifteen mixed conifer forest sites in California exposed to a wide range of N deposition inputs (Table 1) were included in this study. Dominant overstory species were ponderosa (*Pinus ponderosa* Laws) or Jeffrey pine (*P. jeffreyi* Grev. and Balf.), and commonly associated species include California black oak

**TABLE 1**
**Throughfall N Deposition at 15 Sites from the Sierra Nevada (SN), San Gabriel Mountains (SGM), and San Bernardino National Forest (SBNF)**

| Site | Coordinates* | | N Deposition |
|---|---|---|---|
| Lassen (SN) | | | 1.5 |
| Rock Creek (SGM) | | | 6.0 |
| Holcomb Valley (SBNF) | 34° 17’ 35.27” | 116° 54’ 43.15” | 6.1 |
| Shaver Lake (SN) | 37° 08’ 33.95” | 119° 15’ 09.10” | 6.7 |
| Camp Osceola (SBNF) | 34° 09’ 43.54” | 116° 51’ 26.77” | 7.5 |
| Vista (SGM) | | | 8.6 |
| Green Valley (SBNF) | 34° 15’ 04.11” | 117° 03’ 02.36” | 8.7 |
| Barton Flats (SBNF) | 34° 09’ 33.48” | 116° 52’ 49.15” | 8.9 |
| Kratka Ridge (SGM) | 34° 20’ 48.60” | 117° 53’ 57.40” | 11.5 |
| Giant Forest (SN) | 36° 34’ 00.84” | 118° 46’ 37.92” | 11.8 |
| Camp Angelus (SBNF) | 34° 08’ 43.87” | 116° 58’ 40.75” | 12.8 |
| Mountain Home (SN) | 36° 13’ 23.28” | 118° 42’ 46.84” | 18.3 |
| Dogwood (SBNF) | 34° 14’ 14.62” | 117° 12’ 18.40” | 33.3 |
| Strawberry Peak (SBNF) | 34° 13’ 49.63” | 117° 14’ 18.31” | 39.2 |
| Camp Paivika (SBNF) | 34° 14’ 23.95” | 117° 19’ 34.22” | 71.5 |

* Coordinate data are not available for all sites. The Lassen site is located in Lassen National Park in the northern Sierra Nevada. Rock Creek, Vista, and Kratka Ridge are located within 3 km of each other in the San Gabriel Mountains northeast of Los Angeles. Nitrogen deposition varies among the three sites because of differing pollutant transport with varying air flow patterns as a function of ridge and canyon orientation and the prevailing northeasterly winds.

(*Quercus kelloggi* Newb.), white fir (*Abies concolor* Gord. & Glend.), and incense cedar (*Calocedrus decurrens* [Torr.] Florin). These sites were located in the Sierra Nevada Mountains (SN) (Lassen, Shaver Lake, Giant Forest, Mountain Home), the San Gabriel Mountains (SGM) northeast of Los Angeles (Rock Creek, Vista, Kratka Ridge), and the San Bernardino National Forest (SBNF) (Holcomb Valley, Camp Osceola, Barton Flats, Green Valley, Camp Angelus, Dogwood, Strawberry Peak, Camp Paivika). The primary use of these forests is recreational. Logging has not occurred since the early 20$^{th}$ century and the 15 sites have not been affected by fire in the last 70 years, except for fires that occurred in the vicinity of some of the SBNF sampling sites in the fall of 2003. In these instances, we did not sample soil close to burned areas. The soil parent material is weathered or decomposed granitic rock, except at Lassen where the soil is of volcanic origin. The soils are generally sandy loam in texture and percent base saturation generally ranges from 70–100%. Nitrogen deposition in throughfall was measured at the sites[12,17,19, and M.E. Fenn, unpublished data] for 2–5 years from 2000–2005 using ion exchange resin throughfall collectors[19]. A description of sampling methods for soil, litter, and needles can be found elsewhere[17,20].

## Calculation of Empirical Critical Loads

Empirical CL were derived using regression analyses (SigmaPlot 4.0, SPSS Inc., Chicago, IL) considering the varying responses of soil, litter, and needle N and C/N ratios and soil pH values to N deposition at the 15 sites considered in this study. Previously published data were used for these analyses[17,21] and soil pH values correspond to unpublished data for samples collected in the San

Bernardino Mountains in June 2006. The following thresholds were derived from the literature, based on the premise that their exceedance would indicate an impairment of ecosystem functioning: 1.2% for foliar N[22], 25 for litter C/N ratio[23,24], and 4.6 for soil pH[25].

## Calculation of Theoretical Critical Loads

The SMB model was used to calculate theoretical critical loads at these sites. According to the Critical Load Mapping Manual (MM)[3], the following equation was used: $CL_{nut}(N) = N_i + N_u + N_{le(acc)}/(1\text{-}f_{de})$. Where $CL_{nut}(N)$ is the critical load of nutrient nitrogen, $N_i$ is the long-term immobilization of N in soil organic matter, $N_u$ is the net removal of N in harvested vegetation and animals, $N_{le(acc)}$ is the acceptable leaching of N, and $f_{de}$ is the denitrification fraction. The $N_{le(acc)}$ term was calculated as the product of the precipitation surplus (Q, $m^3$ $ha^{-1}$ $year^{-1}$) by the acceptable N concentration ($[N]_{acc}$, eq $m^{-3}$). Following MM prescriptions, $N_i$ and $[N]_{acc}$ were set to 1 kg N $ha^{-1}$ $year^{-1}$ and 0.2 mg N $l^{-1}$, respectively, although calculations were also performed using 3.5 kg N $ha^{-1}$ $year^{-1}$[26] and 3 mg N $l^{-1}$[8], respectively. To avoid negative Q values that are often observed in Mediterranean systems, this term was calculated from the long-term average flow volume of a perennial stream (USGS Monitoring Station no. 11063680) draining the Devil Canyon/Camp Paivika watershed (~1600 $m^3$ $ha^{-1}$ $year^{-1}$)[27]. Similarly, $N_u$ was considered to be 0 as recommended by the MM for long-term net uptake of unmanaged forests. Finally, $f_{de}$ was considered to be 0.1, as corresponds to sandy loam soils[26].

## Results and Discussion

The gradient in N deposition at the study sites ranged from a relatively pristine site in the northern Sierra Nevada (1.5 kg N $ha^{-1}$ $year^{-1}$) to heavily polluted sites in the San Bernardino National Forest in southern California with deposition rates as high as 33.3–71.5 kg N $ha^{-1}$ $year^{-1}$ (Table 1). Evidence of disturbance of the N cycle of the Mediterranean mixed conifer forest by increased N deposition was found when comparing the values recorded for the different parameters along the N deposition gradient (Table 2). Strong significant relationships were found between N deposition and the parameters related with the biological responses to N enrichment (Table 2): needle % N content ($r^2 = 0.67$), litter C/N ratio ($r^2 = 0.82$) and litter % N ($r^2 = 0.83$). Mineral soil-related parameters showed either a weaker relationship (C/N ratio, $r^2 = 0.39$) or no relationship at all (total N) in relation to N enrichment.

Threshold values from the literature were applied to the empirical equations of this study to calculate empirical CL (Table 2). Thresholds of 1.2% foliar N[22] and a litter C/N ratio of 25[23] result in empirical CL of 26 and 54 kg N $ha^{-1}$ $year^{-1}$, respectively. These CL values are much higher than expected from previous findings in southern California and the southern Sierra Nevada (approximately 15 kg N $ha^{-1}$ $year^{-1}$ based on nitrate leaching)[17,28]. Therefore, 15 kg N $ha^{-1}$ $year^{-1}$ was included in the empirical equations to derive the thresholds for the different parameters with significant relationships (Table 2). This leads to critical values (CV) of 1.1 for foliar % N, 1.0 for litter % N, and 35.2 for litter C/N ratio. The CV for foliar N agrees with the critical level for foliar N (1.1% N) determined experimentally for ponderosa pine[29]. The empirical litter C/N ratio (35.2) was much higher than the threshold of 25 proposed in the literature, reflecting the generally high ratios found in the mixed conifer forests. Seven of the eight sites included in the analysis had C/N ratios ranging from 29 to almost 40.

**TABLE 2**
**Calculation of Empirical Critical Loads (CL) and Critical Values (CV)***

| | Parameter | Response | CL $R^2$ | Empirical CL (kg $ha^{-1}$ $year^{-1}$) | CV when N Deposition is 15 kg $ha^{-1}$ $year^{-1}$ | CV Derived from the Literature |
|---|---|---|---|---|---|---|
| Nutrient N | % foliar N | + | 0.67 | 26 | 1.1 | 1.2[22] |
| | % litter N | + | 0.83 | na | 1.0 | |
| | Litter C/N ratio | – | 0.82 | 54 | 35 | 25[23] |
| | Mineral soil C/N ratio | – | 0.39 | na | 24 | |
| | Mineral soil % N | ns** | na** | na | na | |
| Acidifying N | pH | – | 0.99 | 26 | 4.8 | 4.6[25] |

* Empirical CL were derived from regression equations of throughfall N deposition vs. the respective parameter, based on data from sites encompassing a range of throughfall N deposition inputs. Thus, the CL values represent the N deposition at which the CV derived from the literature are predicted to occur in mixed conifer forests of southern California.

** ns, not significant; na, not applicable.

A strong relationship between mineral soil pH and N deposition was also found when the six SBNF sites ($r^2$ = 0.99) were considered, suggesting that in addition to the N enrichment processes, N deposition is also causing soil acidification in these ecosystems. This is an unexpected finding considering the high base saturation of these soils, commonly ≥90% base saturation, except in the most polluted sites[20,30], which should be enough to balance potential shifts in soil pH as is the case in other Mediterranean ecosystems[31]. After 50–60 years of elevated N deposition inputs to the San Bernardino National Forest, the high buffering capacity of these soils has apparently been overcome to varying degrees, leading to soil acidification. In an analysis of 251 European sites [25], nitrate leaching greater than 10 kg N $ha^{-1}$ $year^{-1}$ only occurred when the soil pH threshold of 4.6 was exceeded (i.e., pH lower than 4.6). According to the empirical fit of our data, this threshold pH would be reached in the SBNF when N deposition equals 26 kg $ha^{-1}$ $yea^{1}$. In addition to N enrichment, CL for N-related acidifying processes should be also considered in these mixed conifer forests. However, because of the Mediterranean climate, N export in low and mid elevation catchments in California would likely only reach 10 kg N $ha^{-1}$ $year^{-1}$ in above average precipitation years[28].

Theoretical critical loads for nutrient N were also calculated using the SMB approach[3]. When the default parameterization of the model for conifer species on a sandy loam soil was used (Table 3), which are very conservative values, a CL value of 1.4 kg $ha^{-1}$ $year^{-1}$ of total N deposition was derived. However, when a new parameterization was carried out considering more realistic values provided in the literature[8,26] for $N_i$ and $[N]_{acc}$, the original estimate was increased by more than six times (8.8 kg N $ha^{-1}$ $year^{-1}$). Both values are well below the N deposition value of approximately 15 kg $ha^{-1}$ $year^{-1}$ that leads to nitrate leaching in these ecosystems. These discrepancies have also been reported for other ecosystems[8]. Many uncertainties remain to be resolved to parameterize the SMB model, particularly in linking the impairment of soil processes with biological impacts[3]. Most notably, experimental and modeling results for the$[N]_{acc}$ term (acceptable N concentration in leachate) in the SMB model do not corroborate the default value of 0.2 mg N $l^{-1}$ as suggested for conifer species[8].

**TABLE 3**
**Calculation of Theoretical Critical Loads for Nutrient N with the SMB Model**

| Parameter | Loss Rate(kg ha$^{-1}$ year$^{-1}$) | |
|---|---|---|
| | Mapping Manual | Revised |
| N immobilization ($N_i$) | 1.0 | 3.5 |
| Denitrification fraction ($f_{de}$) | 0.1 | 0.1 |
| Acceptable N leaching ($N_{le(acc)}$) | 0.3 | 4.8 |
| **$CL_{nut}$(N)** | **1.4** | **8.8** |

Another issue of concern for forests, and Mediterranean forests in particular, is that the SMB model does not consider plant nutrient uptake ($N_u$) in wild or recreational areas. However, forest management practices, prescribed fire, or wildfire events would have a large impact on this parameter and, therefore, on CL calculations. For instance, fire would likely produce a sharp increase in N CL because N uptake would be large in the early stages of regrowth, and the ecosystem may not reach steady state if forest fires are not controlled. Also of importance for Mediterranean forests is mobilization of N during wet-season precipitation and runoff events leading to seasonal patterns of increased risk of $NO_3^-$ leaching, and the large interannual variations of those risks derived from drought cycles that may last 4–7 years.

In summary, a syndrome encompassing a wide array of ecosystem responses to increased N deposition was found along with robust empirical relationships relating N deposition to plant N uptake, soil fertility, and soil acidification. When threshold values from the literature for parameters indicative of N excess (e.g., foliar N or litter C/N), were applied to the empirical CL equations of this study, this yielded CL values 1.7–3.6 times higher than expected based on nitrate leaching data. Thus, preliminary new thresholds for those parameters are proposed for these Mediterranean ecosystems as a result of this work. Similarly, the theoretical CL from the SMB model was much lower than the field evidence suggests. Further research is needed to derive the thresholds for critical parameters related to the impairment of mixed conifer forest ecosystems in California and to develop or parameterize models that can more realistically simulate critical loads for these ecosystems. In future work, we will also evaluate the effects of harvesting, and varying fire intervals and prescriptions on CL determinations for N as a nutrient.

## ACKNOWLEDGMENTS

Benjamin S. Gimeno was funded by a grant from the State Secretary for Universities and Research of the Spanish Ministry of Education and Science. In addition, this research was partially funded by NSF grant DEB 04-21530. We thank Diane Alexander for laboratory analyses and Tim Blubaugh and Tony Davila for help with field sampling and maintenance of the throughfall monitoring network.
